# Complete Genome Sequence of the *Sesbania* Symbiont and Rice Growth-Promoting Endophyte *Rhizobium* sp. Strain IRBG74

**DOI:** 10.1128/genomeA.00934-13

**Published:** 2013-11-21

**Authors:** Matthew B. Crook, Shubhajit Mitra, Jean-Michel Ané, Michael J. Sadowsky, Prasad Gyaneshwar

**Affiliations:** Department of Agronomy, University of Wisconsin—Madison, Madison, Wisconsin, USAa; Department of Biological Sciences, University of Wisconsin—Milwaukee, Milwaukee, Wisconsin, USAb; BioTechnology Institute and Department of Soil, Water, and Climate, University of Minnesota, St. Paul, Minnesota, USAc

## Abstract

*Rhizobium* sp. strain IRBG74 is the first known nitrogen-fixing symbiont in the *Agrobacterium/Rhizobium* clade that nodulates the aquatic legume *Sesbania* sp. and is also a growth-promoting endophyte of wetland rice. Here, we present the sequence of the IRBG74 genome, which is composed of a circular chromosome, a linear chromosome, and a symbiotic plasmid, pIRBG74a.

## GENOME ANNOUNCEMENT

*Rhizobium* sp. strain IRBG74 was originally isolated in the Philippines from the root nodules of the aquatic legume *Sesbania cannabina*, which is used as a green manure in wetland rice production (1). This bacterium forms nitrogen-fixing root nodules with at least eight different *Sesbania* species (2). More importantly, *Rhizobium* sp. strain IRBG74 can infect rice endophytically, improving plant growth, health, and yields (1, 3), making it a good model system for determining the mechanisms of *Rhizobium*-cereal interactions.

Phylogenetic analysis indicated that *Rhizobium* sp. IRBG74 likely represents a new species in the *Rhizobium*/*Agrobacterium* group, making it the first known instance of a naturally occurring strain in this clade that is capable of forming nodules and fixing nitrogen with a legume (2).

For whole-genome sequencing, 100-bp paired-end libraries were generated from *Rhizobium* sp. strain IRBG74, which was previously marked with mTn*5*ss*gusA20* (2). Sequencing was done using Illumina’s Phusion-based library kits according to their protocols (Illumina, Hayward, CA) and sequenced on Illumina GAIIx machines at the National Center for Genome Resources (Santa Fe, NM). Base calling was done according to the manufacturer’s protocols. The insert sizes averaged 309 nucleotides (nt), and 90,615,496 reads were obtained, comprising approximately 300× coverage of the genome. Read assembly was performed *de novo* using ABySS (http://www.bcgsc.ca/platform/bioinfo/software/abyss/), resulting in 26 contigs. Several *k*-mers were run, and the best resulting assembly was chosen based on assembly contiguity statistics, the placement of a subset of high-quality read pairs in the assembly with correct spacing, and orientation. Potential contig junctions were predicted based on the conservation of synteny with two reference genomes (those of *Agrobacterium tumefaciens* C58 and *Agrobacterium* sp. strain H13-3) and an analysis of contig ends. These predicted contig junctions were resolved by PCR. The complete genome (5,464,982 bp) consists of a circular chromosome (2,844,565 bp, 59.30% G+C content), a linear chromosome (2,035,452 bp, 59.29% G+C content), and a symbiotic plasmid, pIRBG74a (584,965 bp, 57.48% G+C content). The automated annotation of coding sequences (CDSs) was performed with AMIGene (4), and predicted genes were functionally annotated as described by Vallenet et al. (5) and Sugawara et al. (6).

The *Rhizobium* sp. strain IRBG74 genome consists of 5,540 predicted CDSs, of which 2,912, 1,939, and 689 are on the circular chromosome, linear chromosome, and plasmid pIRBG74a, respectively. Additionally, 2 *rrn* operons and 41 tRNA loci were identified on the circular chromosome, and 2 *rrn* operons and 13 tRNA loci were identified on the linear chromosome.

Plasmid pIRBG74a, a *repABC*-family plasmid, contains many of the *nod*, *nif*, and *fix* genes involved in symbiosis. These results are consistent with those of earlier studies (1) and show that *Rhizobium* sp. IRBG74 is a naturally occurring unique species in the *Rhizobium*/*Agrobacterium* clade that likely obtained nodulation capabilities by acquisition of the pIRBG74a symbiosis plasmid and possibly the loss of the tumor-inducing plasmid present in *Agrobacterium tumefaciens*. The availability of this genome sequence will help determine the mechanisms by which *Rhizobium* sp. IRBG74 forms endophytic and growth-promoting associations with rice, an important cereal crop.

### Nucleotide sequence accession numbers.

The genome sequences have been deposited in NCBI GenBank under the accession no. HG518322 (circular chromosome), HG518323 (linear chromosome), and HG518324 (pIRBG74a).
